# 

**Published:** 1863-07

**Authors:** 


### CHICAGO MEDICAL JOURNAL.

**Terms, $2.00 per Annum.**

### CONTENTS OF THE JULY NUMBER.

PAGE.

ORIGINAL COMMUNICATIONS.

A Case of Pyramidal Cataract. By E. S. Holmes, M. D., of Chicago.... 289

SELECTED.

On Force. From a Lecture by Prof. Tyndall, before the Royal Institution........................................................................ 293

Iodine: Its Actions, Normal and Abnormal, upon the Human Body. Being a Review upon this subject, made by Drs. Schueller and Herman. Translated from Constatt's Jahresbericht.............................. 303

The Physiology of Mormonism. By Charles C. Furley, M. D., Assistant Surgeon United States Army .................................................. 307

EDITORIAL AND MISCELLANEOUS ................................................ 310

